# Lower body negative pressure identifies altered central vein characteristics without accompanying changes to baroreflexes in astronauts within hours of landing

**DOI:** 10.1038/s41598-024-51643-1

**Published:** 2024-01-12

**Authors:** C. J. Mastrandrea, D. K. Greaves, J. K. Shoemaker, A. P. Blaber, P. Arbeille, R. L. Hughson

**Affiliations:** 1 Schlegel-UW Research Institute for Aging, Waterloo, ON Canada; 2 University of Waterloo, Waterloo, ON Canada; 3 School of Kinesiology and Department of Physiology and Pharmacology, University of Western Ontario, London, ON Canada; 4 Department of Biomedical Physiology and Kinesiology, Simon Fraser University, Burnaby, BC Canada; 5 Unite Médecine Physiologie Spatiale, CERCOM, Faculté de Médecine-Université de Tours, Tours, France

**Keywords:** Physiology, Cardiology

## Abstract

Cardiovascular deconditioning and altered baroreflexes predispose returning astronauts to Orthostatic Intolerance. We assessed 7 astronauts (1 female) before and following long-duration spaceflight (146 ± 43 days) with minimal upright posture prior to testing. We applied lower body negative pressure (LBNP) of up to − 30 mmHg to supine astronauts instrumented for continual synchronous measurements of cardiovascular variables, and intermittent imaging the Portal Vein (PV) and Inferior Vena Cava (IVC). During supine rest without LBNP, postflight elevations to total peripheral resistance (TPR; 15.8 ± 4.6 vs. 20.8 ± 7.1 mmHg min/l, p < 0.05) and reductions in stroke volume (SV; 104.4 ± 16.7 vs. 87.4 ± 11.5 ml, p < 0.05) were unaccompanied by changes to heart rate (HR) or estimated central venous pressure (CVP). Small increases to systolic blood pressure (SBP) and diastolic blood pressure (DBP) were not statistically significant. Autoregressive moving average modelling (ARMA) during LBNP did not identify differences to either arterial (DBP → TPR and SBP → HR) or cardiopulmonary (CVP → TPR) baroreflexes consistent with intact cardiovascular control. On the other hand, IVC and PV diameter-CVP relationships during LBNP revealed smaller diameter for a given CVP postflight consistent with altered postflight venous wall dynamics.

## Introduction

Long-term unloading of the cardiovascular system in microgravity environments causes cardiovascular deconditioning. Orthostatic intolerance (OI) due to impaired blood pressure (BP) regulation is a known health risk when returning from spaceflight, having been first described in the early 1960s when the pilot of the Mercury-Atlas 9 could not complete a head-up tilt test after only 34-h of flight^1^. Meck and colleagues provided the first data on greater incidence of OI after long-duration spaceflights^2^, and Lee et al. summarized data from 85 Shuttle and ISS astronauts who completed lower body negative pressure (LBNP), tilt or stand tests at different times after return to Earth^3^. They reported that on the day of landing the majority (52 of 56) of Shuttle astronauts could complete the orthostatic test, compared to only 2 of 6 astronauts following missions on the ISS (data not differentiated by sex). Additional data from the same study also suggested slower postflight recovery after long-duration flights. Two of 8 male astronauts developed orthostatic hypotension during a 3-min stand test completed 18–36-h after 6-month missions on the ISS^4^. NASA now considers postflight OI an acceptable risk of spaceflight^5^, as research-led development of techniques including on-orbit exercises, return-associated oral and intravenous saline, and compression garments, assist in BP regulation^6,7^.

Certain astronauts appear be at greater risk of OI, possibly due to individualized physiological adaptations to microgravity and subsequent return to upright posture on Earth. Altered baroreflex control following spaceflight could contribute to reduced orthostatic tolerance immediately following landing. Following short-duration Shuttle flights, astronauts with poor orthostatic tolerance had impaired vagally-mediated cardiac baroreflexes^8^, lower levels of sympathetic activation and peripheral vasoconstriction^9,10^ and, in women, greater reduction in blood volume^10^. Analyzing responses to postflight tilt across short-duration and long-duration missions, an index derived from diastolic BP and stroke volume predicted OI^3^. Factors during spaceflight that might contribute to OI are complex and interactive. In addition to gravitational unloading, overall levels of physical activity are reduced despite exercise countermeasures^11–13^.

Changes to central vein compliance in returning astronauts may also exacerbate orthostatic intolerance^14^. In the absence of head-to-foot gravity vector, fluid shifts increase cross-sectional area of the jugular and femoral vein by 40% compared to preflight supine posture^15^. Persistent venous dilation in a rat model may ‘reset’ venous wall tone^16^ with increased tangential wall stress and sympathetic innervation, leading to decreased distensibility at lower intraluminal pressures with dampened myocyte contractility, potentially affecting cardiopulmonary baroreflex sensitivity via alteration of venous return to the heart. These relationships have never been assessed following spaceflight.

Rapid cardiovascular deconditioning increases the incidence of orthostatic intolerance with only hours of exposure to head-down bed rest^14^ and recovery quickly follows return to upright posture^17^. Astronauts who had impaired baroreflex responses during 9–10-day space missions did not exhibit baroreflex impairment when tested on landing day following Shuttle missions^18^ but were upright and walking for several hours before testing. There was no indication if these astronauts exhibited any symptoms of OI following flight. To better understand potential changes to cardiovascular control to orthostatic challenge we took advantage of long-duration astronauts landing in a supine position on the Shuttle then being transported to the laboratory in a supine position with minimal exposure to head-to-foot gravity vector as they transitioned to the crew transport vehicle. For Soyuz landed astronauts, they were upright during transport back to crew quarters but then spent the night in bed and were collected from their bed and carried to the laboratory in supine position before testing. We hypothesized that baroreflex control following spaceflight would be altered, with enhancement of cardiopulmonary and attenuation of arterial baroreflex responses during LBNP in the hours following landing. We limited post-flight stimuli to normal head-up position that might provoke blood volume and vascular regulatory adaptations in order to better test this hypothesis. Following on previous findings of attenuated flow reduction in the splanchnic and femoral veins during LBNP post bed rest that were associated with OI^19^, we hypothesized that reductions to portal vein diameters during LBNP would be attenuated post-flight.

## Methods

### Participants

Seven ISS astronauts (1 female, 47 ± 4 years of age, mean mission length: 146 ± 43 days) were recruited following informed consent procedures approved by the University of Waterloo Office of Research Ethics (ORE#11763), European Space Agency Medical Review Board, Japanese Space Agency Medical Review Board, NASA Human Research Medical Review Board and Johnson Space Center Institutional Review Board (previously known as Committee for the Protection of Human Subjects) (NASA7116301606HR), in accordance with the Declaration of Helsinki. All participants signed an informed consent form before participating and were made aware that they could withdraw from the study at any time.

Testing was performed at either the Johnson Space Center (Houston, TX), Kennedy Space Center (Cape Canaveral, FL), Dryden Flight Research Center (Edwards, CA), or Gagarin Cosmonaut Training Center (Star City, Russia) depending on mission parameters. Preflight data were collected approximately 30-days before launch. Four of the astronauts launched aboard the space Shuttle, and three launched on the Soyuz. All astronauts fluid-loaded immediately prior to landing, some wore lower body compression garments during return to Earth^20,21^ that were removed prior to testing. Shuttle-landed astronauts returned to Earth in a supine position with only very brief periods of head-up posture during transition to a gurney on the crew transport vehicle. They were delivered to the research laboratory and moved to the experiment bed whilst remaining supine. Astronauts who returned to Earth on Soyuz were transported to Star City with upright posture and walking; testing for one astronaut was delayed by 24 h due to weather. After their first overnight sleep, the experiment team transported the astronauts to the laboratory in a supine position to avoid upright posture on the day of experimentation. The time between landing and testing in Shuttle participants was 3 h and 30 min ± 22 min. The time between landing and testing in Soyuz participants was 25 h and 25 min and 28 h 25 min for two Soyuz astronauts while the other was 49 h and 33 min (Table 1).

**Table 1:** Exact delays between landing and the start of data collection for each astronaut investigated in this study.

Landing vehicle	Landing to testing (hours and minutes)
Shuttle	3 h and 59 min
Shuttle	3 h and 8 min
Soyuz	25 h and 25 min
Shuttle	3 h and 6 min
Soyuz	28 h and 28 min
Soyuz	49 h and 33 min
Shuttle	3 h and 37 min

### Experimental protocol

**Figure 1:**
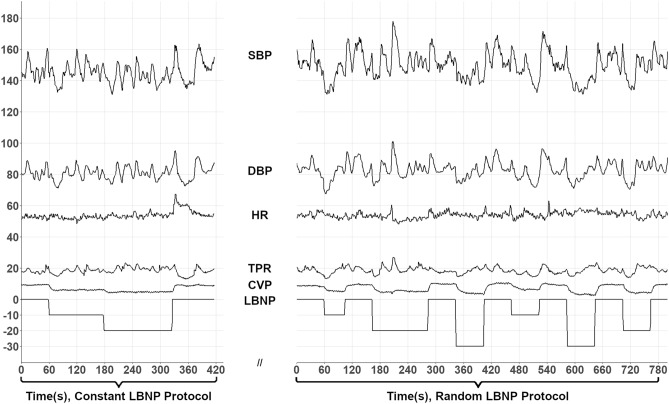
Example preflight data acquisitions from Constant (Left) and Random (Right) LBNP Protocols in one subject showing SBP (mmHg), DBP (mmHg), HR (bpm), TPR (mmHg.min/l), CVP (cmH_2_O), and LBNP pressure (mmHg). Data collection periods in Constant LBNP were nominally 5-min for instrumentation and baseline then 2-min at each LBNP including ultrasound scan times.

Physiological data were collected during Constant and Random LBNP protocols. The Constant Protocol was always performed first, following supine instrumentation and 5-min of baseline data collection. The Constant Protocol consisted of four periods each nominally lasting 2-min including time for ultrasound imaging: Baseline, − 10 mmHg LBNP, − 20 mmHg LBNP, and Recovery. Following this, another rest period preceded the Random Protocol, which itself consisted of a sequence of LBNP steps as shown in Fig. 1, reaching a maximum negative pressure of − 30 mmHg. At these levels of LBNP, estimated redistribution of blood volume to the extremities would be approximately 400–500 ml^22^. The Random Protocol was required for analysis with our chosen Auto Regressive Moving Average investigative technique.

### Equipment

Physiological variables were recorded at 1000 Hz through a PowerLab system (ADInstruments, Australia). For those astronauts landing in Russia, finger pressure was recorded with a Finapres device (Finapres Medical, Netherlands), with subsequent modelling of continual cardiac output (${\dot{\text{Q}}}$) values via Beatscope software (Finapres Medical, Netherlands). When calculated, stroke volume (SV) was obtained by dividing ${\dot{\text{Q}}}$ (l/min) by heart rate (HR). For all other astronauts, finger pressure was recorded alongside modelled cardiac output (Finometer, Finapres Medical, Netherlands). HR was calculated from the electrocardiogram (Model 7830A, Hewlett-Packard, USA). Central venous pressure (CVP) was estimated from the dependent arm technique via a pressure transducer (TransStar 60″ Single Monitoring Kit, Smith Medical ASD, Inc., USA) attached to a saline-flushed 21G cannula placed in the right antecubital fossa^23^. Astronauts were positioned with a custom foam wedge into a right tilt to maintain a continuous column of blood from the catheter to the right atrium, and the position of the pressure transducer was established by a laser level at the right atrium referenced to the anterior axillary line. LBNP was achieved using a custom-built LBNP box that allowed foot support, with pressure monitored via a pressure transducer (ADInstruments, Australia). We calculated total peripheral resistance (TPR) from mean arterial pressure (MAP), CVP and ${\dot{\text{Q}}}$ from the equation:

$$TPR\left( {{\text{mmHg}}\;\min /{\text{l}}} \right) = \frac{{MAP\left( {{\text{mmHg}}} \right) - CVP\left( {{\text{mmHg}}} \right)}}{{\dot{Q}\left( {{\text{l}}/\min } \right)}}$$

Measurements of the diameters of the Inferior Vena Cava (IVC ø) and Portal Vein (PV ø) were performed on images obtained via standard ultrasound techniques using either a Sonosite (Sonosite, USA) or Logiq Book (GE Healthcare, USA) ultrasound device. Images of both the IVC and PV were obtained at 0 mmHg, − 10 mmHg and − 20 mmHg LBNP during the first three LBNP step-changes of the Constant Protocol. Ultrasound landmarks in the proximity of the IVC and PV were confirmed during the preflight collections and were visualized at postflight collections to ensure the measurement of vein diameters at the same location. Astronauts were instructed to calmly hold their breath at normal inspiration without performing a Valsalva during acquisitions to reduce the impact of respiratory cycle on venous dimensions. No ultrasound measurements were taken during the Random Protocol.

### Data analysis

We obtained beat-by-beat values for RR interval, SBP, DBP, MAP, ${\dot{\text{Q}}}$ and respiration rate. CVP was recorded as the mean of values obtained between each heartbeat. For datasets without Finometer-recorded ${\dot{\text{Q}}}$, Finapres finger waveforms were calibrated to simultaneous manual sphygmomanometer measurements performed by a trained researcher, down sampled to 100 Hz and processed with Beatscope software (Finapres Medical Systems, Netherlands) to produce modelled brachial blood pressures and cardiac output, then time-synced to data obtained for all other variables. Beat-by-beat data were interpolated to achieve equally spaced 1-s sampling for all variables prior to further analyses. Venous diameters were taken from a mean of 3 cross-sectional acquisitions perpendicular to each vessel, acquired during a relaxed breath hold with the interpreter blinded to participant and/or the timing of the acquired images.

Baseline data are reported as mean values of the last 30 s of an initial baseline data collection period prior to commencing LBNP (Table 2). For steady-state analyses, we averaged measurements of all variables over the last 30 s of each LBNP step, allowing for identification of cardiovascular responses to differing LBNP intensities. Vein diameter/CVP relationships were derived from vein diameter (mm) and CVP (cmH_2_O) measurements recorded simultaneously during the baseline, − 10 mmHg and − 20 mmHg LBNP steps of the Constant Protocol.

**Table 2:** Group resting cardiovascular variables in the supine position prior to the commencement of LBNP (n = 7).

Resting variable	Measurement technique	Preflight	Postflight	p-value
Heart rate (bpm)	ECG R-R intervals	51.8 ± 7.2	51.9 ± 6.0	0.9528
Systolic blood pressure (mmHg)	Modelled from finger waveform	120.6 ± 13.9	129.1 ± 15.6	0.0629
Diastolic blood pressure (mmHg)	Modelled from finger waveform	74.3 ± 8.6	83.6 ± 11.8	0.0511
Cardiac output (l/min)	Modelled from finger waveform	5.5 ± 1.6	4.6 ± 1.1	0.0800
Stroke volume (ml)	Modelled from finger waveform	104.4 ± 16.7	87.4 ± 11.5*	0.0109
TPR (mmHg min/l)	Calculated from MAP, CVP & ${\dot{\text{Q}}}$	15.8 ± 4.6	20.8 ± 7.1*	0.0158
CVP (cmH_2_O)	Pressure transducer waveform	11.6 ± 1.1	13.0 ± 3.8	0.3966
IVC diameter (cm)	Ultrasound imaging	1.5 ± 0.3	1.4 ± 0.4	0.0969
PV diameter (cm)	Ultrasound imaging	0.7 ± 0.3	0.7 ± 0.1	0.4245

Data provided as mean ± standard deviation. p-value provided for comparisons between preflight and postflight values.

*p < 0.05.

Spectral analyses were performed on interpolated Random LBNP protocol data. We saw consistent patterns of power identified with discrete Fourier transform, with the greatest power at the sixth harmonic of both input signals (LBNP and CVP). Subsequent reconstitution of transformed signals, with differing numbers of harmonic frequencies, were then compared to original signals following calculation of mean square errors. After consideration of mean square error, absolute gains and spectral patterns, it was concluded that the first 9 harmonics would allow accurate analyses of changes in the LBNP, CVP and TPR signals, a methodology for signal analysis validation previously described by Hughson et al.^24^. In addition to spectral analyses, autoregressive moving average (ARMA) investigations were also performed on Random protocol data. ARMA represents a linear time-invariant system, allowing for analysis of multiple input variables for a single output signal^25,26^. For these experiments, two sets of input and output signals were analysed to further quantify the cardiovascular responses to LBNP (Input: CVP and DBP, Output: TPR; or Input CVP and SBP, Output HR). Computation was performed using custom written ARMA Matlab software^26^. From the resulting step-changes (the modelled change in output signal for a sustained change of 1 unit Input signal), values for the plateau, and time taken to reach 95% of the plateau, were calculated and compared. All signal interpolation, spectral, and ARMA analyses were performed using Matlab software (MathWorks, USA). Due to poor CVP signal quality during the random LBNP protocol for two participants, we were only able to use data from five of the seven participants for spectral and ARMA analyses.

### Statistics

Differences in baseline preflight and postflight CVP, TPR, HR and ${\dot{\text{Q}}}$ were investigated with paired t-tests. Two-way matched repeated measures ANOVAs (Flight Status x LBNP) were used to test for significant differences at differing LBNP intensities between preflight and postflight for HR, SBP, DBP, ${\dot{\text{Q}}}$, SV, TPR, CVP, IVC ø, PV ø and PV velocity. If deemed necessary, Bonferroni multiple comparisons were used when required. Repeated measures correlations were performed (CVP vs TPR, CVP vs SV, CVP vs IVC ø, and CVP vs PV ø) using the package ‘rmcorr’ from RStudio, and provided us with the ability to investigate responses within the group without violating assumptions of interdependence^27^. Subsequently, ARMA results were analysed with Wilcoxon matched pairs, testing significant differences in the plateau and time to 95% of plateau values. Statistical analyses were performed with Prism software (GraphPad, USA) and R-(RStudio, USA), with statistical significance reported when p < 0.05. All data are presented as mean ± SD unless otherwise stated.

## Results

### Resting pre-test postflight variables

We identified elevations to resting TPR immediately following spaceflight and prior to LBNP, accompanied by close-to-significant increases in SBP and DBP, and reductions to ${\dot{\text{Q}}}$, with marked reduction in SV (Table 2). There were no differences to resting HR or CVP.

### Responses to LBNP

**Figure 2:**
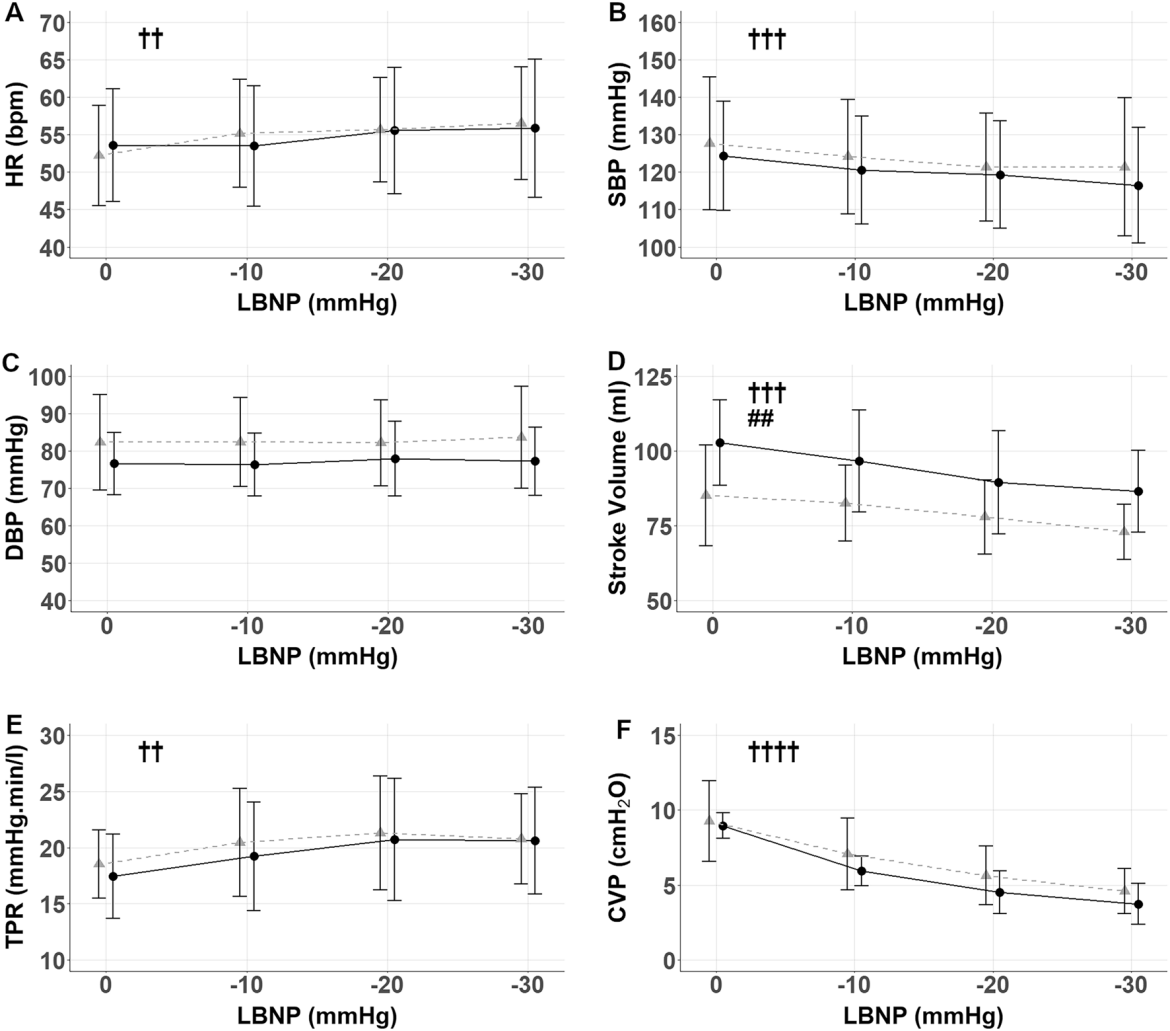
Group changes in cardiovascular variables with increasing negative pressure, displayed here as mean group values during the last 30 s of each period without LBNP and each LBNP intensity. Therefore, the LBNP ‘0’ includes data from all periods without LBNP during the constant and random LBNP protocol. Preflight (black circles with solid line) and postflight (grey triangles with dashed line). Data shown as mean ± SD in 7 participants for: HR (**A**), SBP (**B**), DBP (**C**), SV (**D**) and in 5 participants for TPR (**E**), and CVP (**F**). ^†^Significant effect of LBNP, ^†^p < 0.05, ^††^p < 0.01, ^†††^p < 0.001, ^††††^p < 0.0001. ^#^Significant effect of spaceflight, ^##^p < 0.01. The slight horizontal offset exists to aid visualisation.

Progressive LBNP intensity resulted in reductions to SBP (p = 0.0008, Fig. 2B), ${\dot{\text{Q}}}$ (p < 0.0001), CVP (p < 0.0001, Fig. 2F), IVC ø (p = 0.0016) and PV ø (p = 0.0007), with reciprocal increases in HR (p = 0.0012, Fig. 2A) and TPR (p = 0.0091, Fig. 2E). SV also decreased with LBNP (P < 0.0001, Fig. 2D) with an additional effect of spaceflight (p < 0.01). DBP was not altered by LBNP before or after spaceflight during our testing (Fig. 2C).

**Figure 3:**
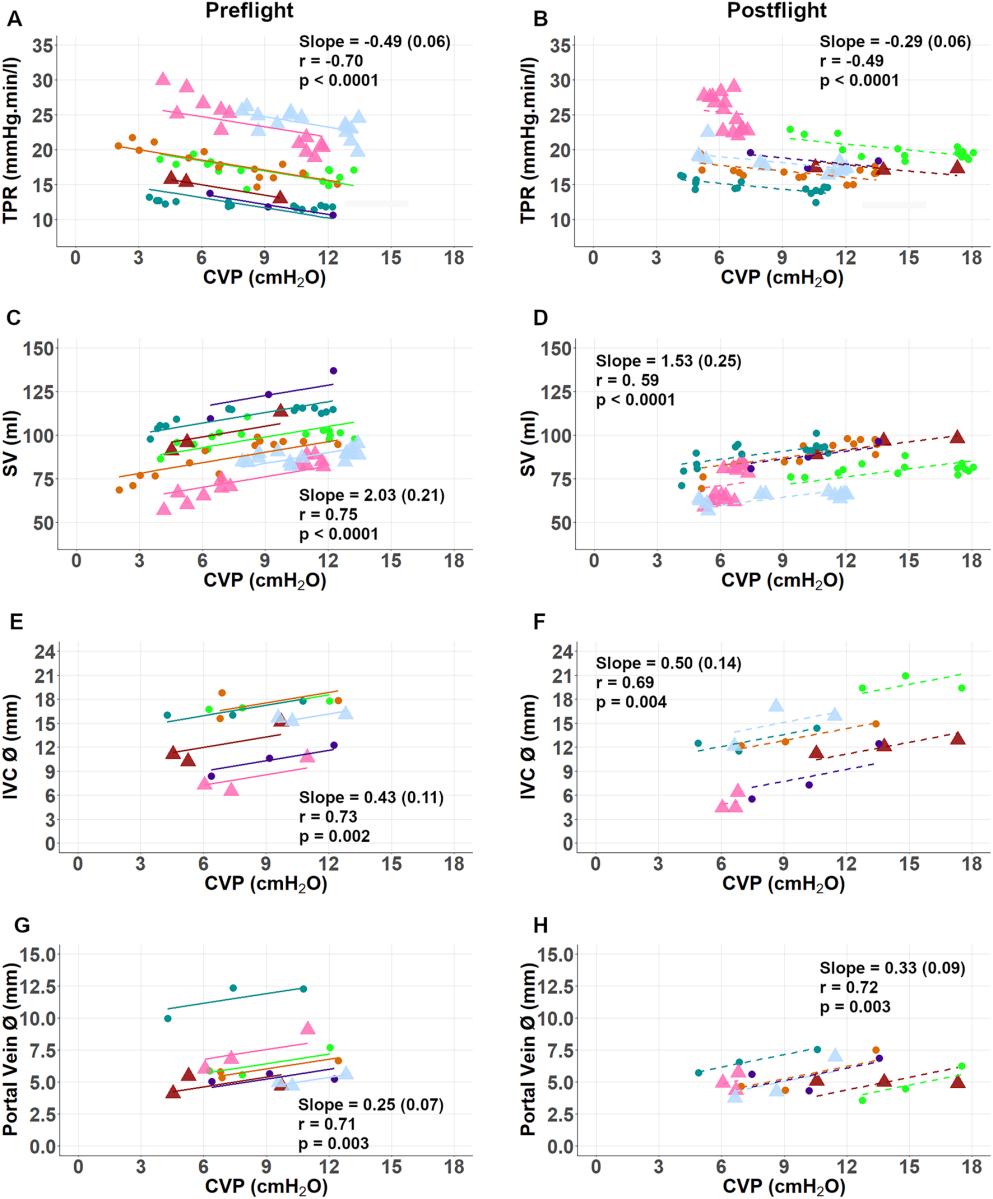
Repeated Measures Correlations for CVP vs TPR (**A** and **B**), CVP vs SV (**C** and **D**), CVP vs IVC ø (**E** and **F**), and CVP vs PV ø (**G** and **H**). Preflight data with solid lines (**A**,**C**,**E**,**G**) left, and Postflight data with dashed lines (**B**,**D**,**F**,**H**) right, are shown as circles representing Shuttle astronauts (black circles with solid line) and triangles representing Soyuz astronauts (black triangles with dashed line). Colour represents individual participants. For 2 of the 7 participants, only data taken from the Constant Protocol is plotted for CVP vs TPR and CVP vs SV, due to poor CVP quality during the Random LBNP Protocol.

Cardiovascular responses expressed as a function of changes in CVP while manipulating LBNP are shown for individual astronauts preflight and postflight with repeated measures correlation in Fig. 3. With reductions in CVP, stimulating the vasoconstrictor arm of the cardiopulmonary baroreflex, TPR increased preflight (Fig. 3A, r = − 0.70, slope coefficient = − 0.49, p < 0.0001) and postflight (Fig. 3B, r = − 0.49, slope coefficient = − 0.29, p < 0.0001). SV positively correlated with CVP, an index of cardiac filling pressure, during both the preflight and postflight sessions (Fig. 3C, r = 0.75, slope coefficient = 2.03, p < 0.0001, and Fig. 3D r = 0.59, slope coefficient = 1.53, p < 0.0001, respectively).

**Figure 4:**
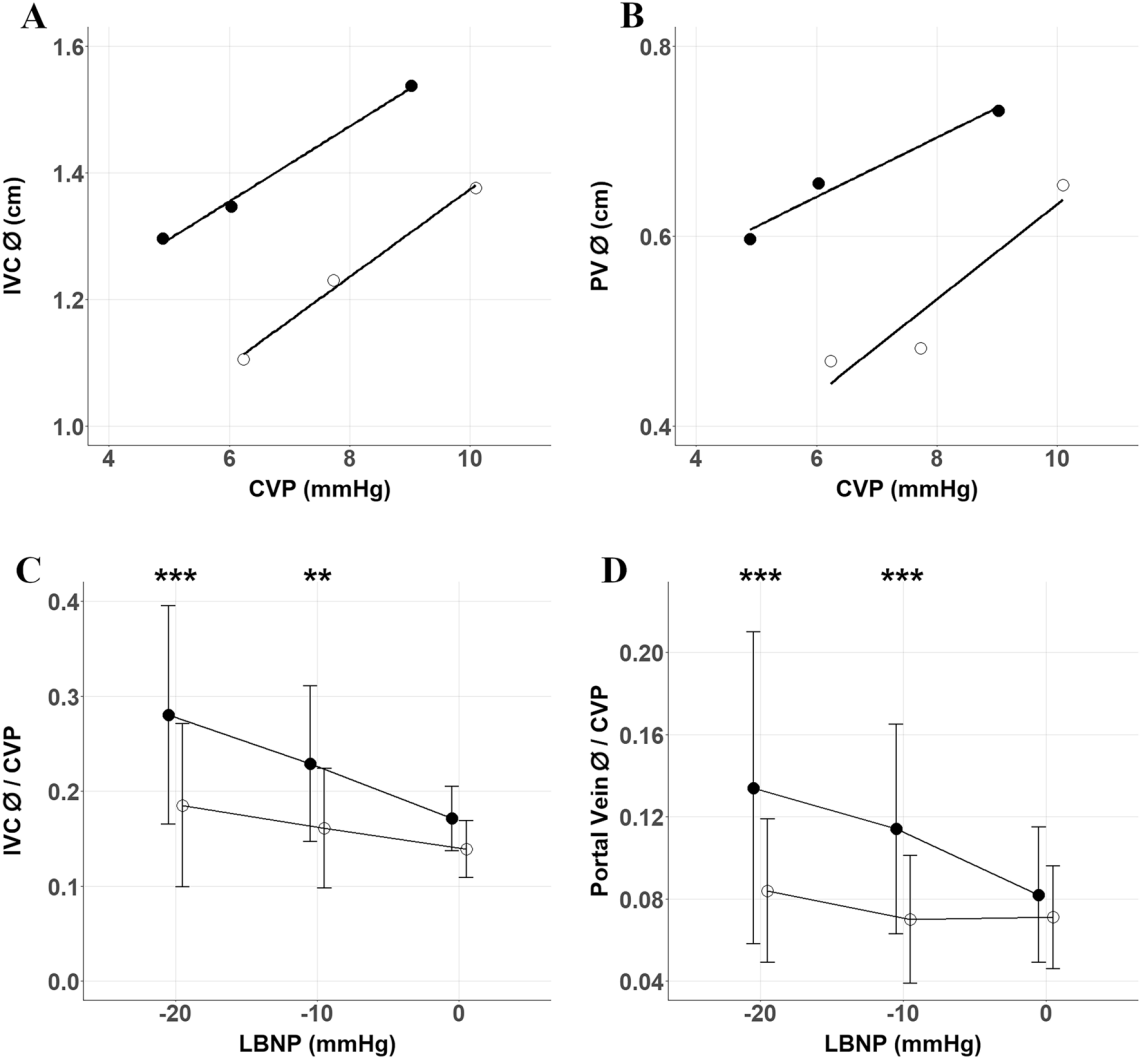
Vein ø shown as a function of CVP for the inferior vena cava (IVC) (**A**) and portal vein (PV) (**B**), and the ratio for IVCø/CVP (**C**) and PVø/CVP (**D**) (mean ± SD) at different LBNP intensities in preflight (black circles) and postflight (open circles). Two-way ANOVA identified significant interaction effects for both veins (p = 0.0268 for IVCø/CVP and p = 0.005 for PVø/CVP). Post-hoc Bonferroni testing identified significant differences between preflight and postflight groups at − 10 mmHg and − 20 mmHg LBNP. **p < 0.01, ***p < 0.001. The slight horizontal offset exists to aid visualisation.

We note positive relationships between CVP and venous volume indicated by IVC diameter (ø) preflight (Fig. 3E, r = 0.73, slope coefficient = 0.43, p = 0.002) and postflight (Fig. 3F, r = 0.69, slope coefficient = 0.50, p = 0.004). Similarly, CVP had a positive relationship with PV diameter preflight (Fig. 3G, r = 0.71, slope coefficient = 0.25, p = 0.003) and postflight (Fig. 3H, r = 0.72, slope coefficient = 0.33, p = 0.003). Assessment of differences in vein diameter/CVP relationships during LBNP were identified from pre- to postflight (Fig. 4 and Table 3). Two-way RM ANOVAs confirmed significant interaction effects (p = 0.0268 for IVCø/CVP Fig. 4C and p = 0.005 for PVø/CVP Fig. 4D), with post-hoc comparisons identifying no differences at LBNP 0 mmHg, but significant differences at − 10 mmHg and − 20 mmHg for postflight compared to preflight measurements for IVCø/CVP and PVø/CVP. Noted elevations to PV velocities between preflight and postflight sessions at all levels of LBNP were close to achieving statistical significance (p = 0.058, Table 3), with no independent effect of LBNP nor interactive effect of LBNP*Spaceflight.

**Table 3:** CVP (mmHg), Vein Diameters (ø**,** cm) and velocities (cm/s) measured at each step LBNP intensity during the Constant LBNP Protocol.

	LBNP = 0mmHg	LBNP = − 10mmHg	LBNP = − 20mmHg
CVP PRE	9.03 ± 0.80	6.03 ± 1.16	4.89 ± 1.26
CVP POST	10.09 ± 2.73	7.73 ± 2.39	6.23 ± 1.95
IVC ø PRE	1.54 ± 0.27	1.35 ± 0.41	1.29 ± 0.38
IVC ø POST	1.38 ± 0.37	1.23 ± 0.56	1.11 ± 0.46
PV ø PRE	0.73 ± 0.25	0.66 ± 0.24	0.60 ± 0.17
PV ø POST	0.65 ± 0.09	0.48 ± 0.08	0.47 ± 0.08
PV velocity PRE	19.1 ± 3.34	17.7 ± 5.31	17.9 ± 4.72
PV velocity POST	20.0 ± 4.56	22.7 ± 3.71	22.1 ± 3.70

Note that the resting CVP values in Table 2 represent those prior to commencement of the experiment, whilst those in Table 3 are pressures recorded simultaneously with the ultrasound collections and are therefore slightly different and incorporate experimental stimuli.

### Dynamic cardiovascular interactions

**Figure 5:**
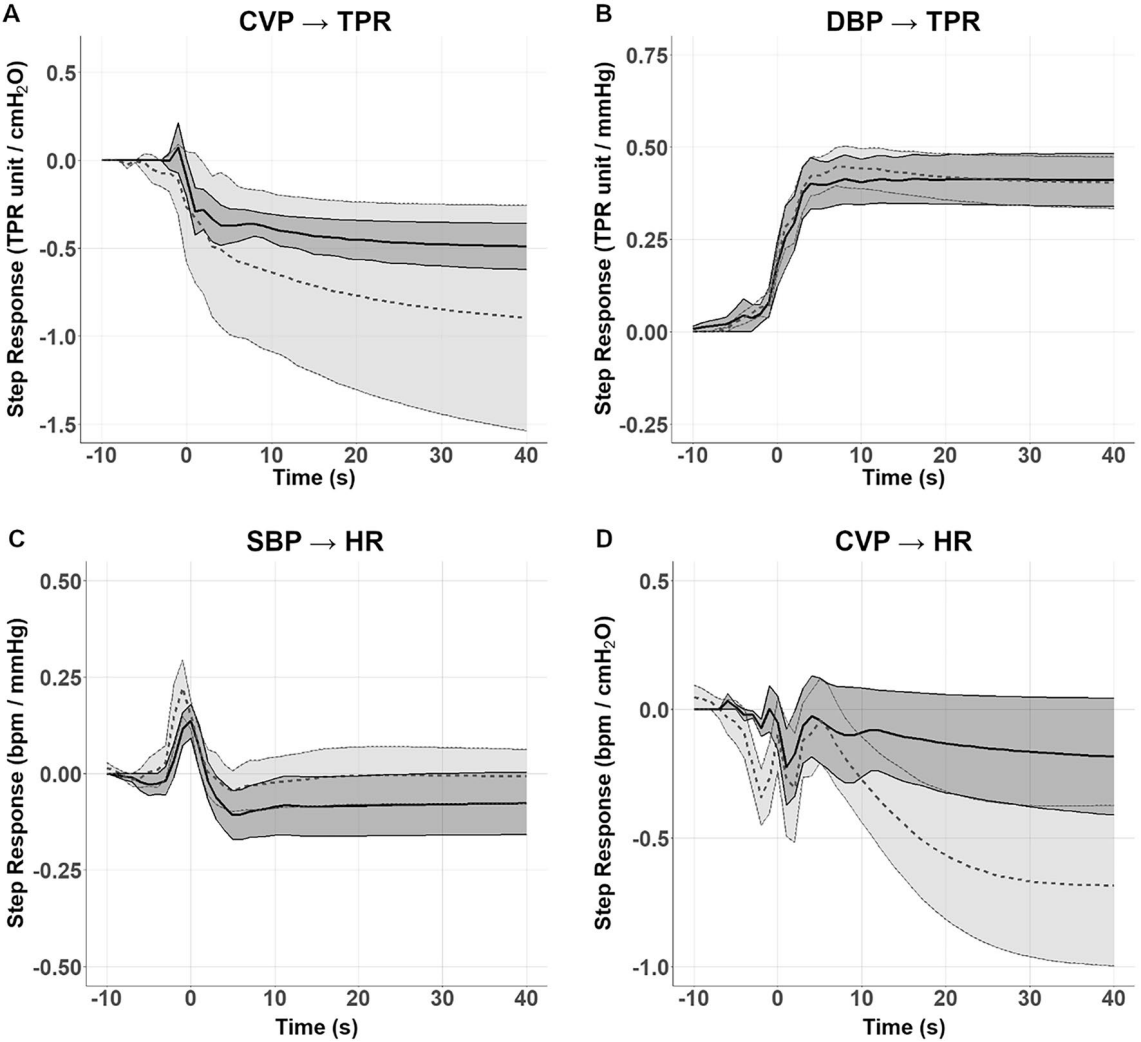
Calculated Step Responses (ARMA analyses) of CVP → TPR (**A**), DBP → TPR (**B**), SBP → HR (**C**) and CVP → HR (**D**). Preflight (solid line and dark shading) and postflight (dashed line and pale shading). No differences in plateau values or time to 95% plateau values were noted.

Cardiopulmonary and arterial baroreflex response characteristics were explored during the Random LBNP Protocol. In the frequency domain, the gain of the cardiopulmonary baroreflex obtained from the relationship of TPR to CVP was no different preflight to postflight (data not shown). These findings were corroborated by ARMA modeling (Fig. 5) that found no differences in absolute changes in TPR to a 1 cmH_2_O increase in CVP (− 0.49 ± 0.29 TPR units/cmH_2_O preflight, − 0.90 ± 1.43 TPR units/cmH_2_O postflight) and time to 95% plateau (15.4 ± 6.8 s preflight, 22.2 ± 10.4 s postflight). Visually, the postflight change in TPR appeared greater than preflight but was driven entirely by one astronaut with small changes in CVP (see cluster of points in upper left quadrant of Fig. 3B). Arterial vascular baroreflex relationship of DBP to TPR showed little change following spaceflight (Fig. 5B), with no difference in plateau values for step response to a 1 mmHg increase in DBP changes (0.41 ± 0.16 TPR units/mmHg preflight, 0.40 ± 0.15 TPR units/mmHg postflight) or time to 95% plateau values (8.6 ± 9.3 s preflight, 4.6 ± 3.1 s postflight).

HR responses to changes in SBP revealed no difference in the plateau values for SBP → HR (− 0.08 ± 0.18 vs − 0.01 ± 0.16 bpm/mmHg), nor the time to 95% of plateau (25.4 ± 16.1s vs 13.2 ± 8.8s bpm/mmHg), preflight and postflight respectively (Fig. 5C). Plateau CVP → HR step responses (Fig. 5D) did not identify significant changes in either plateau (0.18 ± 0.51 bpm/cmH_2_O preflight, − 0.69 ± 0.70 bpm/cmH_2_O postflight, p = 0.3) or the time to 95% of plateau values. Three of the five tested astronauts had greater postflight HR responses to changes in CVP leading to visual differences, but these were not statistically significant.

## Discussion

The incidence of orthostatic intolerance on return from long-duration spaceflight is greater than that after shorter duration flights^2,3^. In this investigation of long-duration spaceflight, we applied submaximal orthostatic-like stresses with mild LBNP to investigate potential changes in cardiac, vascular and reflex control mechanisms. Uniquely, the astronauts were exposed to no, or minimal, upright posture between landing and test sessions by transporting in supine position from Shuttle landings or carrying supine from bed to the laboratory after overnight supine posture for Soyuz landings. This approach limited physiological readaptation to orthostatic stresses following spaceflight, minimizing stimulation of neurohumoral mechanisms promoting the expansion of blood volume and the concomitant priming of cardiovascular responses to such challenges. Contrary to our hypothesis, and to previous bed rest findings^28,29^, the estimate of CVP was unchanged rather than lower following spaceflight. Instead, we unexpectedly observed smaller IVC and PV diameters relative to the changes in CVP during exposure to mild LBNP after spaceflight. We also found that SV was reduced postflight despite no change in estimated CVP. Additionally, and unlike observations of cardiovascular deconditioning after bed rest^29,30^ or other investigations of spaceflight^31,32^, there were no significant changes in HR, SBP, DBP or baroreflex responses in supine rest or during mild LBNP from preflight to postflight.

### Venous responses to LBNP after spaceflight

This is the first study to explore adaptations of the Inferior Vena Cava and Portal Vein and their impact on an astronaut’s cardiovascular responses postflight. With spaceflight, directly measured CVP is elevated on assuming a launch position and during the high g period of launch but decreases rapidly on entry to microgravity^33^ as has also been observed with parabolic flight^34^. In bed rest studies, CVP is elevated on initial movement to head-down position but decreases within the first few hours^35,36^. This decline probably reflects “creep” of venous smooth muscle^37^ since it occurs before plasma volume is significantly reduced, and was speculated to underlie impaired orthostatic tolerance after only 4-h in the head-down position^14^. Further, it was observed after 4- or 28-h head down bed rest that estimated CVP was lower on return to supine position and remained lower throughout an LBNP challenge^28,29^ unless fluid loading was introduced^38^. However, in the current study, where estimated CVP was not different at rest or during LBNP following spaceflight, a non-quantified pre- and post-return fluid loading and other factors, including hormonal responses, could have impacted total blood volume affecting our measurements. Nevertheless, alternative adaptations of the venous system should also be considered.

Central veins including the portal vein are enlarged during spaceflight^39^ as a consequence of enhanced transmural pressure, despite no elevation to CVP^33^. Chronic in-flight dilation of the IVC and PV might underlie the changes we observed to the relationship between vein diameter and estimated CVP at each LBNP step (Fig. 4). We found a marked reduction in IVC diameter/CVP and PV diameter/CVP at both − 10 mmHg and − 20 mmHg, but not at 0 mmHg. Central veins are often regarded as relatively passive, adjusting their diameter to the current distending pressure. In each of pre-flight and post-flight testing, this characteristic that defines venous compliance is observed during LBNP (Fig. 4A and B) but we observed a shift identified by the significant reduction in diameter/CVP ratio (Fig. 4C and D). Alterations in venous properties and innervation have been identified under conditions that chronically manipulate venous distending pressure. Chronic distension of rat saphenous vein caused multiple adaptations including increased diameter with maintained wall thickness, increased tangential wall stress, reduced distensibility, smooth muscle cell hyperpolarization that could dampen myocyte contraction, and evidence of increased sympathetic neural input following selective blockade with tetrodotoxin^16^. Therefore, the attenuated post-flight venous responses to LBNP-induced changes in CVP we identify in our participants here may relate to a relative vessel hypertrophy, altered sympathetic activation, or reduced vasomotor responses of the PV similar to those observed in the femoral artery during head-down bed rest^19^.

### Cardiac and vascular responses following spaceflight with LBNP

Responses of the primary cardiovascular variables in the current study contrast somewhat with the responses at rest and to LBNP measured 1–2 days after returning from 8 to 20 days in space by Baisch et al.^40^. Most notably, while we found an elevation in TPR during pre-LBNP supine rest, Baisch et al. reported a significant reduction. They also noted a lower SBP while we observed a trend to elevated SBP and DBP corresponding to the higher TPR. Baisch et al. studied cardiovascular responses to LBNP at − 15, − 30 and − 45 mmHg. They did not report statistical comparisons at the two lower levels of LBNP that would have corresponded to LBNP used in the current study but did find significant elevation in HR and reduction in SV and Q postflight at − 45 mmHg LBNP. In postflight testing, we observed no change in HR despite lower SV at rest and during LBNP. It is not known if the differences between studies resulted from the longer duration of spaceflight in the current study that allowed for adaptations and enhanced countermeasures, patterns of physical activity or other countermeasures during flight, or if greater exposure to upright posture prior to testing in their study might have contributed to the contrary findings. Baisch et al. observed clear evidence, through body impedance measurements, that reduced intravascular volume played a critical role in the postflight cardiovascular response to LBNP, while autonomic regulation of cardiovascular responses was not changed^40^. Reduced intravascular volume appears consistent with our observation of smaller IVC and PV diameter relative to CVP during postflight LBNP testing, but we do not have blood volume measurements and no data on fluid loading regimes. Our findings of reductions to SV postflight despite little change in estimated CVP, with small and statistically insignificant differences to TPR and blood pressure, might suggest cardiac-specific mechanisms, such as reduced cardiac mass observed in short-duration spaceflights^31^ or impaired diastolic untwisting observed after 18-days head-down bed rest^41^. However, changes in cardiac function with longer duration spaceflight are not clear, with some astronauts showing improved cardiac function during maximal exercise^42^.

Following the 16-day Neurolab mission, Levine et al. described the cardiovascular and autonomic responses while supine and during a 10-min 60-deg head-up tilt test^43^. During supine rest, SV was smaller post flight as we found, but the elevation in TPR was not significant which contrasts to our observations. The non-significant elevation of TPR while supine and in head-up tilt in the Neurolab study occurred with significant elevations in muscle sympathetic nerve activity^43^. Elevated resting TPR in the current study was probably accompanied by increased sympathetic vasoconstriction. The greater orthostatic challenge of head-up tilt in Neurolab compared to that of LBNP in the current study was associated with significant postflight elevation in HR which we did not observe. Their findings of little change to postflight blood pressures during tilt matched our findings during LBNP.

### Dynamic and reflex responses

The ARMA modeling approach to investigate cardiovascular control considered the potential simultaneous effects of different inputs on the output variable of interest. For the dynamic regulation of TPR, the model included the cardiopulmonary baroreflex effects of changes in CVP on TPR together with the arterial baroreflex effects of changes in DBP on TPR. There was no difference in the gain of either of these reflex loops on comparing preflight to postflight models for the cohort as a whole. The cardiopulmonary baroreflex was also assessed in the frequency domain with similar observations of no effect of spaceflight. These results were not expected, as previous ground-based studies identified augmented cardiopulmonary baroreflexes using similar ARMA methodology following 4-h head-down bed rest^29^. Enhanced cardiopulmonary baroreflex was also found from the relationship between CVP and forearm vascular resistance after 7-days head-down bed rest^44^. Enhanced cardiopulmonary baroreflex probably also contributed to observations after the 16-day Neurolab mission of increased muscle sympathetic nerve activity in direct proportion to the reduction in cardiac stroke volume during 60-degree upright tilt^43^, and increased norepinephrine spillover at baseline and during LBNP^45^. Our contradictory results may relate to a resetting of cardiopulmonary baroreflexes after longer-duration spaceflight following changes in pressure–volume relationships and/or elevated central blood volume. Additionally, CVP was not measured during the Neurolab mission, and CVP-SV relationships may have been altered^41^.

Our identification of one astronaut with considerably different CVP → HR and CVP → TPR responses postflight is in keeping with heterogeneous individual orthostatic responses postflight^9^. This one astronaut accounts for a large proportion of the wide variability in the postflight responses seen in Fig. 5A and D. Additionally, these responses were accompanied by small central venous diameters and minimal changes to vessel size during LBNP. Therefore, the enhancement of cardiopulmonary baroreflexes in this individual was potentially accompanied by maximal stimulation of central veins throughout testing; the diameter of both the IVC and PV in this astronaut were smaller postflight. The lack of any reduction in vein diameter may suggest an almost maximal stimulation of these vessels even at baseline. Subsequently, it may be that this individual would have had a low tolerability to tilt-table or formal orthostatic intolerance testing at the time of testing, but the minimal LBNP intensities used during this study were too low to elicit pre-syncopal symptomatology. SBP → HR responses were not affected by flight in this individual.

Dynamic regulation of HR was modeled by ARMA with the input of the arterial baroreflex from changes in SBP to HR, and with the potential effects of changes in CVP to HR. Previously, in the male astronauts of the current study, we reported reduced spontaneous arterial baroreflex responses that related changes in R-R interval to changes systolic BP only when the astronauts were seated upright during paced-breathing^32^. Here, while testing under the challenge of mild to moderate LBNP in our population of 6 men and 1 woman, there were no changes in the SBP to HR relationship following spaceflight, even though SV was lower postflight. Previously after short-duration spaceflights arterial baroreflex gain was reduced even in supine rest^8,18^. These results could relate to the very stressful short missions with limited time for countermeasures, disrupted sleep and no indication of fluid-loading prior to return to Earth. With longer ISS missions, cardiovascular control appears to stabilize near Earth supine values while in space^46^, but some astronauts have greater increases in resting HR reflecting lower vagal activity^32^.

### Consideration of protocol and limitations

Investigations in astronauts are limited by the number of available participants for physiological research. Nevertheless, conducting such research is important and provides the scientific community with precious insight into physiological changes occurring during spaceflight. We acknowledge that the small sample size in this investigation may have been inadequate to achieve statistical significance for some outcomes. Other limitations of this study include the maximal LBNP of − 30 mmHg, resulting in relatively mild fluid shifts that challenge the cardiovascular system less than that incurred during a lying-to-standing postural change. The decision to limit LBNP to this low level was made for safety reasons in place at the time of the study and we were unable to expose our participants to greater magnitudes of LBNP. However, we did see changes to estimated CVP, and were subsequently able to identify differences in venous properties, investigate autonomic baroreflexes, and identify variability within individuals. Differences in post-landing test session timing between astronauts flown in the Shuttle vs Soyuz required differences in posture with periods upright after a Soyuz landing, that may have masked spaceflight-induced changes. The additional time delay occurred for three of our participants, during which time a degree of blood volume recouperation could have occurred. However, it is known that autonomic changes associated with spaceflight persist for a number of days following short missions^47^, and therefore any impact of this brief delay to our results was likely to be minimal especially when the astronauts were carried horizontal from their bed to the laboratory.

Methodological considerations included our inability to directly measure CVP during these experiments due to astronaut safety considerations and the invasiveness of placing central catheters immediately following spaceflight. The dependent arm technique relies on establishing a continuous column of blood from the transducer, through the catheter to the central vein^23^. The presence of characteristic pulsatility in the pressure signal in combination with careful positioning of the transducer at right heart level by a laser level minimized the risk of aberrant values. Changes in the tissue properties of the vessel wall, skin and sub-cutaneous tissues surrounding the venous catheter, nor their potential influence on absolute CVP pressures were not assessed. However, there was no obstruction from the central column of blood to the pressure transducer recording these values, and we believe any tissue changes would therefore not influence absolute CVP values measured in our study. Measuring absolute CVP with this technique is not perfect and must be taken into consideration when interpreting our findings; however, changes in CVP should be reflected by our method. Additionally, determination of cardiac output and SV using Modelflow algorithms could have been influenced by increases in arterial stiffness following 6-months of spaceflight^12^. Estimated SV is smaller at older ages for a given arterial pulse wave^48^; however, Modelflow has never been compared to a standard method before and after spaceflight, and comparisons of pre-flight with inflight^12^ focused on very different physiological conditions. Finally, the constant LBNP protocol preceded the random LBNP protocol for each participant at both timepoints. A test order effect might have occurred but it was not evident in data pooled across the test types as in Fig. 3.

## Conclusion

The ability to study astronauts within hours of returning to Earth, especially prior to resuming upright posture, provided us with a unique opportunity to test cardiovascular responses before the re-establishment of compensatory physiological responses to 1G. We identified changes to resting supine cardiovascular variables that corroborate previously identified elevations to global sympathetic tone following spaceflight. Our investigations of dynamic cardiovascular responses to incremental and random LBNP challenge did not support our initial hypotheses of enhanced cardiopulmonary and diminished arterial baroreflexes in our participants. However, we did identify one astronaut with enhancement of both CVP → HR and CVP → TPR responses postflight, reinforcing the heterogeneity with some astronauts more susceptible to stresses of upright posture after spaceflight. Finally, we found important alterations to IVC/CVP and PV/CVP relationships during LBNP, which could suggest central vein hypertrophy or enhanced sympathetic innervation in these vessels. It may be that changes in venous pressure/volume relationships influence maintenance or elevation of CVP in central venous capacitance vessels during postflight orthostatic challenges.

## Data Availability

The datasets used and/or analyzed during the current study are available from Dr. Richard Hughson (hughson@uwaterloo.ca) on reasonable request.

## Abbreviations

ARMA: Autoregressive moving average
BP: Blood pressure
CVP: Central venous pressure
DBP: Diastolic blood pressure
HR: Heart rate
IVC: Inferior vena cava
ISS: International space station
LBNP: Lower body negative pressure
MAP: Mean arterial pressure
OI: Orthostatic intolerance
PV: Portal vein
Q̇: Cardiac output
SV: Stroke volume
SBP: Systolic blood pressure
TPR: Total peripheral resistance
